# Remote Ischemic Perconditioning is Effective After Embolic Stroke in Ovariectomized Female Mice

**DOI:** 10.1007/s12975-013-0318-6

**Published:** 2014-01-04

**Authors:** Md Nasrul Hoda, Kanchan Bhatia, Sherif S. Hafez, Maribeth H. Johnson, Shahneela Siddiqui, Adviye Ergul, Syed Kashif Zaidi, Susan C. Fagan, David C. Hess

**Affiliations:** 1 Department of Medical Laboratory, Imaging and Radiologic Sciences, Georgia Regents University, Augusta, GA USA; 2 Department of Neurology, Georgia Regents University, 1120 15th St, CA 1014, Augusta, GA 30912 USA; 3 Department of Medicine, Georgia Regents University, Augusta, GA USA; 4 Department of Physiology, Georgia Regents University, Augusta, GA USA; 5 Biostatistic, Georgia Regents University, Augusta, GA USA; 6 Program in Clinical and Experimental Therapeutics, College of Pharmacy, University of Georgia, Augusta, GA USA; 7 Charlie Norwood VA Medical Center, Augusta, GA USA; 8 Center of Excellence in Genomic Medicine Research, King Abdul Abdulaziz University, PO Box 80216, Jeddah, 21589 Kingdom of Saudi Arabia

**Keywords:** Stroke, Embolic stroke, Remote ischemic conditioning, IV-tPA thrombolysis

## Abstract

Remote ischemic conditioning is neuroprotective in young male rodents after experimental stroke. However, it has never been tested in females whom remain at higher risk of stroke injury after menopause. We tested remote ischemic perconditioning therapy (RIPerC) at 2 h after embolic stroke in ovariectomized (OVX) female mice with and without intravenous tissue plasminogen activator (IV-tPA) treatment. We assessed cerebral blood flow (CBF), neurobehavioral outcomes, infarction, hemorrhage, edema, and survival. RIPerC therapy with and without IV-tPA improved the CBF and neurobehavioral outcomes and reduced the infarction, hemorrhage, and edema significantly. Late IV-tPA alone at 4 h post-stroke neither improved the neurobehavior nor reduced the infarction but aggravated hemorrhage and mortality in OVX mice. RIPerC therapy prevented the increased mortality during late IV-tPA. Our study demonstrates for the first time that RIPerC therapy is effective in OVX females.

## Introduction

While neuroprotective agents in acute stroke clinical trials have failed, a promising therapeutic approach is the modulation of endogenous protective mechanisms by remote ischemic per- and post- conditioning (RIPerC and RIPostC), a safe, inexpensive and feasible therapy for stroke. RIPerC and RIPostC are effective in reducing infarct size and improving functional outcomes in different rodent models of stroke [1]. However, all these preclinical studies to date have been done in young male rodents. Despite some promising clinical trials of limb conditioning in cardiac patients, there are still no preclinical studies in females. The progression of post-stroke pathophysiology primarily depends upon the dynamics of cerebral blood flow (CBF) and may be sexually dimorphic [2–4]. A therapy that works in males after stroke may not be effective in females [2, 4–6]. Moreover, there is an increasing interest in therapies that can reduce reperfusion injury. Therefore, the Stroke Academic Industry Roundtable (STAIR) recommends preclinical studies in both sexes of animals in clinically relevant models and therapies that can reduce reperfusion injury [7].

An embolic model of stroke in animals better approximates the dynamics of CBF changes, infarct progression, and maturation as it occurs in humans and also allows combination studies with intravenous tissue plasminogen activator (IV-tPA) [2, 3, 8], the only FDA approved reperfusion therapy. We reported that RIPerC therapy after embolic stroke in young male mice increases ‘early’ CBF, subsequently leading to neurobehavioral benefits and neuroprotection with and without IV-tPA [9]. However, the therapeutic potential of RIPerC therapy has never been tested in female rodents. Such preclinical data is necessary before RIPerC can be translated into humans and before clinical trials of RIPerC and RIPostC can be initiated in acute stroke.

In this work, we tested RIPerC therapy at 2 h after embolic stroke in ovariectomized female (OVX) mice with and without ‘late’ IV-tPA at 4 h, that is, near the end of the ‘clinical time window’. OVX mice are frequently used as a model of post-menopausal women whom remain at higher risk of stroke and poor outcomes [5]. We reported that non-OVX young females are comparatively resistant to embolic stroke injury, which is lost after ovariectomy [10]. Such OVX mice are also inclined to hemorrhagic transformations (HT) after embolic stroke. In this study, we used OVX mice 2-month post-ovariectomy and at the age of ∼6 months. We hypothesized that RIPerC alone will remain effective in OVX mice as in males due to early CBF improvement and will reduce the detrimental effects of late IV-tPA.

## Materials and Methods

### Animals, Experimental Groups, and Procedures

The Institutional Animal Care and Use Committee of Georgia Regents University (GRU) approved all animal procedures prior to the start of the experiments as per the National Institute of Health guidelines. A total of 140 C57BL/6 J wild type OVX mice (20 ± 2 weeks old; 8 ± 1 weeks post-OVX; Jackson Laboratory, Bar Harbor, Maine) housed in GRU's AAALAC accredited facility were used in the following experiments. As reported earlier by us [9] and based on two RIPerC (no vs. yes) by two tPA (no vs. yes) design, the following two experiments were performed in four groups, eMCAO + Veh, eMCAO + RIPerC, eMCAO + tPA, and eMCAO + RIPerC + tPA. In **Experiment I**, early relative CBF changes out to 6 h were performed on six animals per group using laser Doppler flowmeter (LDF; PeriFlux 5001, PeriMed Inc., Sweden) as reported earlier and in the related supplementary information [9]. Representative images were also obtained from each group using PIM3 laser Doppler scanner (PeriMed), and the cerebral perfusion of the two hemispheres were compared [9–11]. Different neurobehavioral tests as discussed below and injury size estimation were performed on all the surviving animals of this cohort. In **Experiment II**, six surviving animals of each group were assigned each for hemorrhagic transformation and edema quantitation.

The grouping, sample size estimation, randomization, and blinding strategies including all other animal procedures and treatments were similar to as reported earlier by us [9–11] and in the related supplementary information. The survival and mortality from both the above experiments was pooled and summarized in Table 1. All the animals were sacrificed 24-h post-stroke. Because of higher death rate in OVX mice [10] and anticipated further increase in the mortality due to late IV-tPA treatment and HT, we reduced the size of the clot and used a 7.0 ± 0.5-mm long clot.

**Table 1:** Survival mortality in the different groups pooled from the two experiments 24 h post-stroke

Experiments/groups	Total # of animals	Survived	Dead
Experiments I and II			
eMCAO+Veh	30	20 (66.7 %)	10 (33.3 %)
eMCAO+RIPerC	30	23 (76.7 %)	07 (23.3 %)
eMCAO+tPA	40	19 (47.5 %)	21 (52.5 %)
eMCAO+RIPerC+tPA	40	28 (70.0 %)	12 (30.0 %)

### Remote Ischemic Perconditioning (RIPerC) Therapy

The noninvasive RIPerC procedures in anesthetized animals have been reported earlier as well as recently and were found effective [12, 13]. At 2-h post-stroke, RIPerC sham procedure in non-conditioned groups (eMCAO + Veh and eMCAO + tPA groups) or RIPerC therapy in the conditioned groups (eMCAO + RIPerC and eMCAO + RIPerC + tPA groups; 250 ± 10 mm Hg × 4 cycles, 10 min each × 10 min interval between cycles) were performed noninvasively under mild isofluorane anesthesia using an exclusive programmable cuff-based conditioner and (Hatteras Instruments, NC, USA).

### Neurobehavioral Assessments

Adhesive tape test to detect sensorimotor deficit was performed at 18–20-h post-stroke with slight modification [14, 15]. Briefly, naïve mice were acclimatized for 3 days prior to surgery. Within maximum time duration of 180 s, the tape removal time was recorded as the function of sensorimotor outcome. Mice were scored 180 s if they failed to remove the tape. Neurological deficit score (NDS) was assessed at 24-h post-stroke and before sacrifice.

### Spectrophotometric Assay of Intracerebral Hemorrhage

Hemoglobin (Hb) content (*n* = 6 per group) in the brain was estimated as reported [16]. Briefly, 20 uL of supernatant obtained from the ipsilateral tissue homogenate (*n* = 6 per group) was mixed with 80 uL of Drabkin's reagent (Sigma Chemical Co.). The 96-well plate was read at 540 nm after 15 min. Hb-content in the brain samples was calculated from the standard curve.

### Estimation of Edema

Brain water content (*n* = 6 per group) was determined as an indicator of edema, as described by Hoda et al [17].

### Statistical Analyses

All the data are expressed as mean ± SD, statistical analyses were performed as reported earlier using SAS 9.3 (SAS Institute Inc., Cary, NC, USA). Briefly, a rank transformation was used prior to analysis where needed to stabilize variance across groups. A two RIPerC (no vs. yes) by two tPA (no vs. yes) ANOVA with interaction was used to analyze ‘within time CBF’, neurobehavioral outcomes, stroke injury, Hb-content, and edema in experiments I and II. In the absence of a significant interaction, the main effects are considered to be additive when combined. The effect of groups on survival mortality was determined using chi-square tests. Statistical significance was determined at *p* < 0.05.

## Results

### RIPerC After Embolic Stroke Improved CBF With and Without IV-tPA

**Fig. 1:**
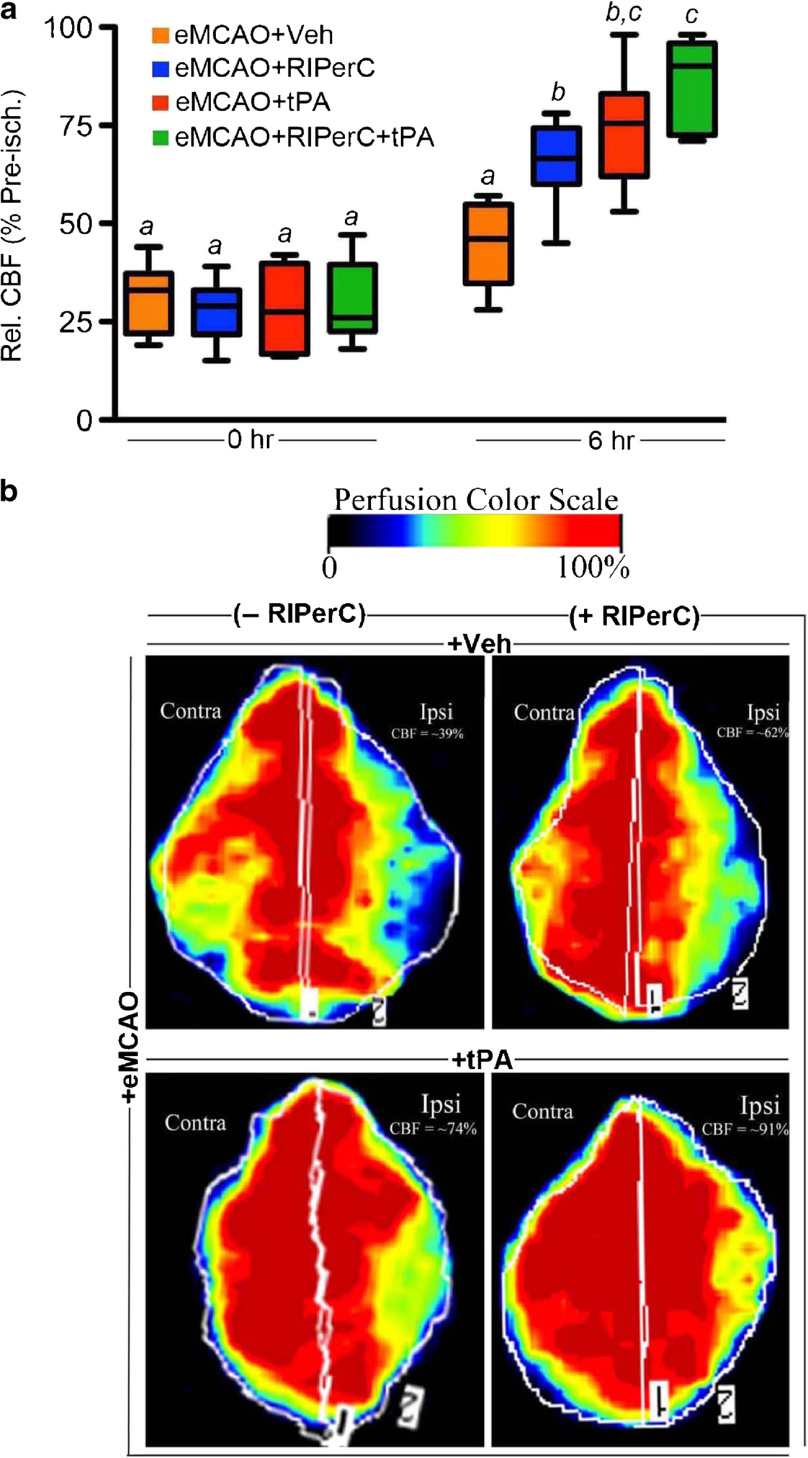
**a** CBF in the different groups measured by laser Doppler flowmeter (PeriFlux 5001) 0 and 6 h post-stroke. The LDF signal was recorded in the MCA territory of the ipsilateral hemisphere semi-continuously before (pre-ischemia) and after stroke. The absolute CBF value was obtained as an average of the values recorded over a period of 10 min at the required time points. The final data was calculated and presented as the percent of the pre-ischemic CBF value. Comparisons between the groups were done ‘within the time point’. All the data were expressed as mean ± SD (*n* = 6). Pairs of means with different letters are significantly different, *p* < 0.05. **b** Representative images of the cerebral perfusion in different groups as detected by laser Doppler perfusion imager (PeriScan PIM3 scanner 6 h post-stroke and 4 h after RIPerC therapy with and without IV-tPA. The values shown in the panels are as compared with their contralateral hemispheres

The immediate post-stroke CBF relative to the pre-ischemic value in the ipsilateral hemisphere, as measured by the LDF, was not significantly different between the groups (Fig. 1a, Experiment I). RIPerC therapy 6 h post-stroke in eMCAO + RIPerC group improved the CBF as compared with RIPerC sham-operated eMCAO + Veh group (RIPerC effect *p* = 0.0040 at 6 h post-stroke). Late IV-tPA therapy also improved the CBF significantly in eMCAO + tPA group (tPA effect *p* < 0.0001 at 6 h post-stroke). RIPerC showed an additive effect in combination with IV-tPA therapy in further improving the CBF since there was no significant interaction between the two treatments (interaction effect, *p* = 0.39 at 6 h post-eMCAO). Moreover, cerebral perfusion imaging by PIM3 laser scanner in the representative animals 6 h after stroke confirmed these changes (Fig. 1b).

### RIPerC After Embolic Stroke Conferred Neurobehavioral Benefits and Neuroprotection With and Without IV-tPA

**Fig. 2:**
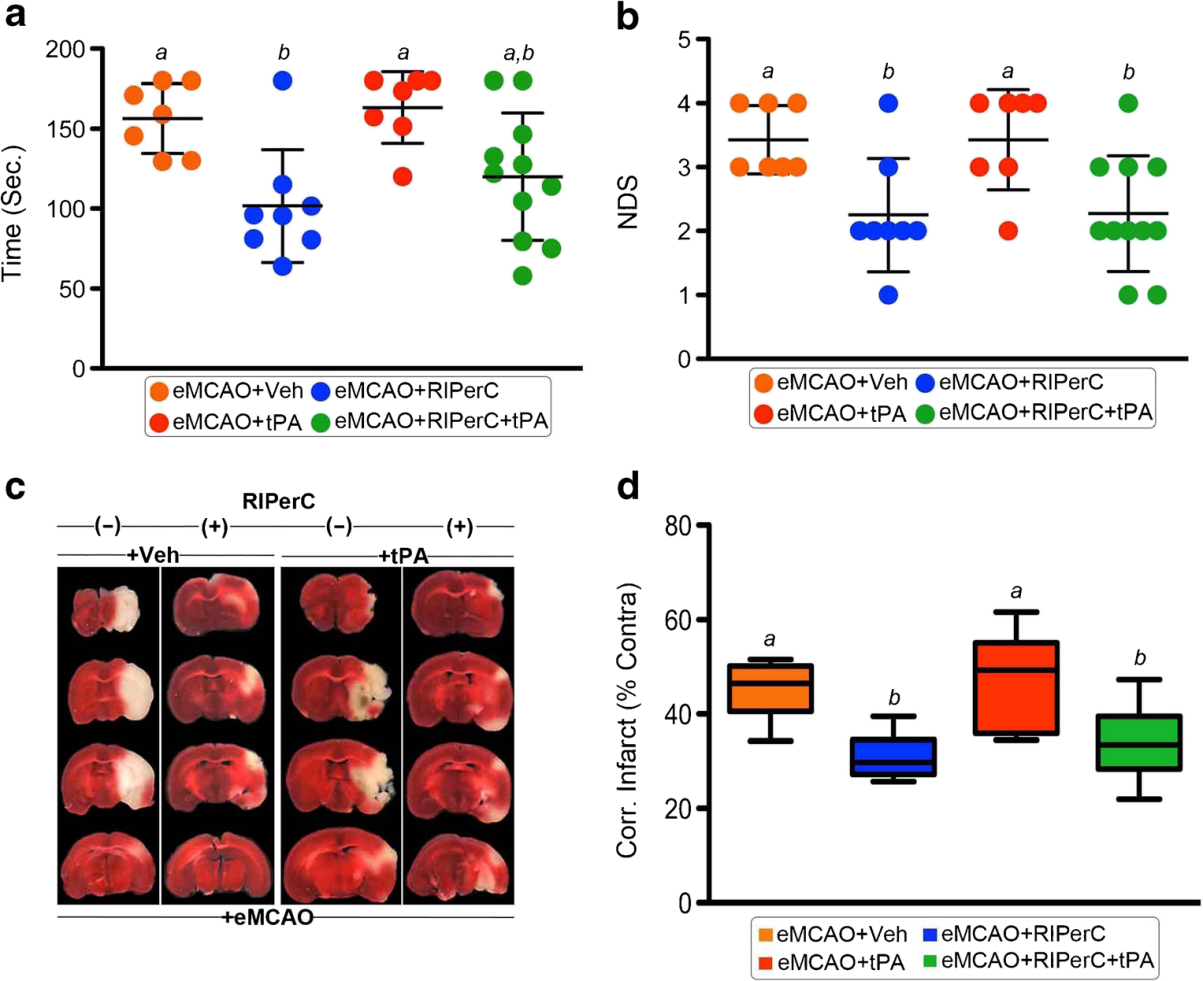
Neurobehavioral and infarction assessments in surviving animals. **a** Sensorimotor function assessed by adhesive tape removal test at 18–20 h post-stroke and **b** neurological deficit score (NDS) as assessed on modified Bederson scale at 24 h post-stroke and before sacrifice. All the data were expressed as mean ± SD. **c** Representative TTC-stained coronal sections and **d** means of the corrected infarct volumes calculated as the percent of their corresponding contralateral sides. Data were expressed as mean ± SD (*n* = as denoted by the number of circles in A and B). Pairs of means with different letters are significantly different, *p* < 0.05

We tested the surviving animals from Experiment I for two different behavioral outcomes at 18–24 h post-stroke (Fig. 2a,b). The main effects of RIPerC on both sensorimotor and NDS outcomes were significant (*p* = 0.0004 and *p* = 0.0003, respectively). There was neither a significant effect of IV-tPA nor a significant interaction between the treatments. RIPerC significantly improved the sensorimotor function in eMCAO + RIPerC group as compared with eMCAO + Veh (101.7 ± 35.2 vs. 156.4 ± 21.9; *p* = 0.014). IV-tPA treatment alone in eMCAO + tPA group did not improve the sensorimotor function significantly as compared with eMCAO + Veh (163.2 ± 22.3 vs. 156.4 ± 21.9; *p* = 0.98). The sensorimotor function of the eMCAO + RIPerC group was significantly better than the eMCAO + tPA group (*p* = 0.0051). On the other hand, combination therapy in eMCAO + RIPerC + tPA group significantly improved the sensorimotor function as compared with eMCAO + tPA (120.0 ± 39.9 vs. 163.2 ± 22.3; *p* = 0.046) but not significantly when compared with eMCAO + Veh (*p* = 0.11). RIPerC significantly improved the neurologic outcome in eMCAO + RIPerC group as compared with eMCAO + Veh (2.25 ± 0.89 vs. 3.43 ± 0.53; *p* = 0.047). IV-tPA therapy alone in eMCAO + tPA group did not improve the neurologic outcome significantly as compared with eMCAO + Veh (3.43 ± 0.79 vs. 3.43 ± 0.53; *p* = 1.0). The neurologic outcome of the eMCAO + RIPerC group was significantly better than the eMCAO + tPA group (*p* = 0.042). On the other hand, combination therapy in eMCAO + RIPerC + tPA group significantly improved the neurologic outcome as compared with eMCAO + Veh (2.28 ± 0.90 vs. 3.43 ± 0.53; *p* = 0.030) and eMCAO + tPA (*p* = 0.030).

Figure 2c,d (Experiment I) shows the representative images of 2,3,5-triphenyltetrazolium chloride (TTC)-stained coronal sections and infarct volumes, respectively. The analysis of effects on the infarct volume reduction indicates that the main effect of RIPerC was significant (*p* < 0.0001), but the effect of IV-tPA and their interaction were not significant (*p* = 0.51 and *p* = 0.70, respectively). RIPerC therapy alone in eMCAO + RIPerC group significantly reduced the injury size as compared with eMCAO + Veh (31.1 ± 4.6 vs. 45.6 ± 6.2; *p* = 0.0035). IV-tPA treatment alone in eMCAO + tPA group did not reduce the injury size as compared with the eMCAO + Veh (46.3 ± 10.5 vs. 45.6 ± 6.2; *p* = 0.99). Combination therapy in eMCAO + RIPerC + tPA group significantly reduced the infarct size when compared with the eMCAO + tPA group (33.8 ± 7.2 vs. 46.3 ± 6.2; *p* = 0.0075) and also as compared with the eMCAO + Veh group (*p* = 0.013), but it was not significantly different when compared with the eMCAO + RIPerC group (*p* = 0.85).

### RIPerC After Embolic Stroke Provided Neurovascular Protection With and Without IV-tPA

**Fig. 3:**
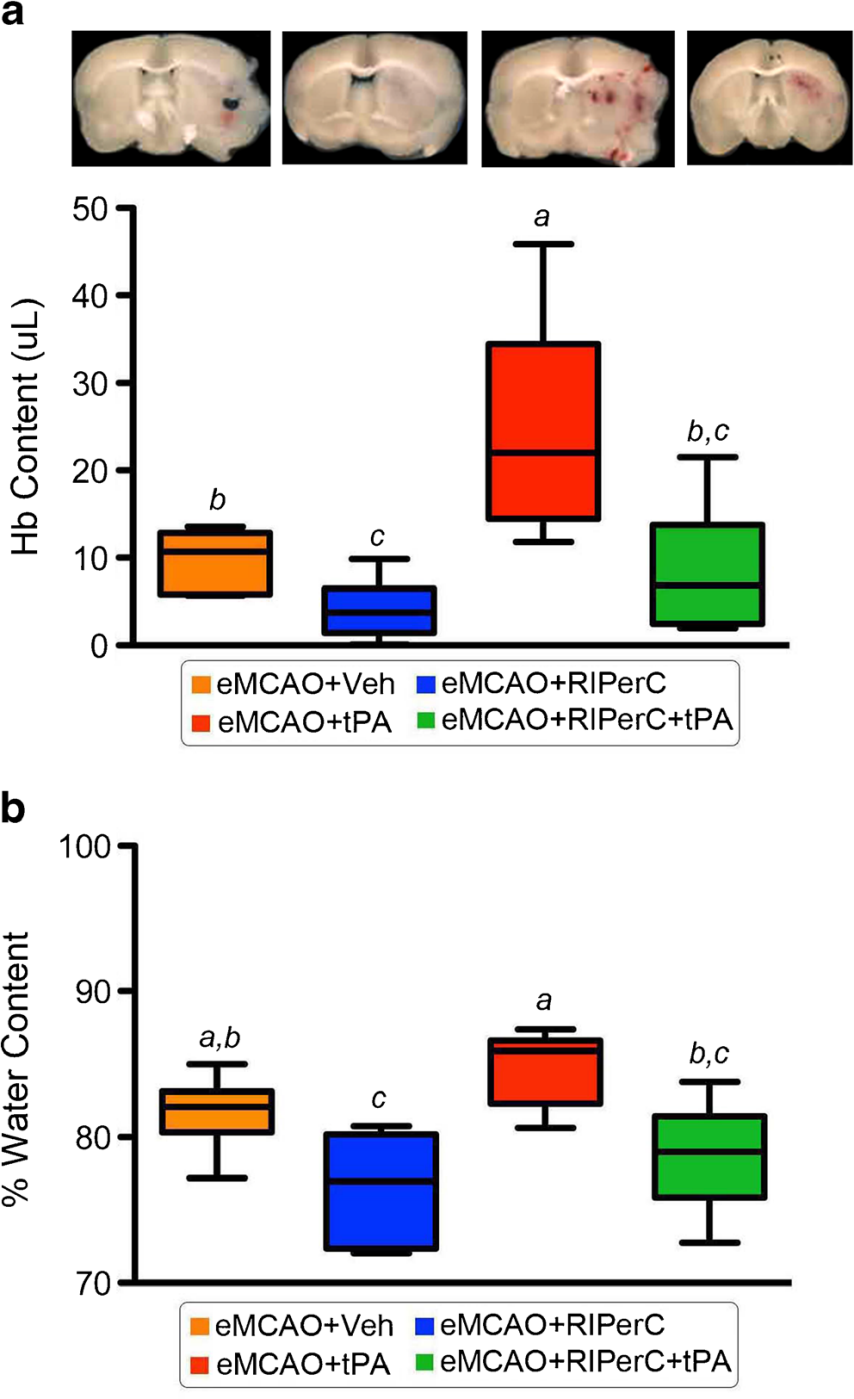
**a** Representative coronal sections from each group showing hemorrhagic transformations and quantitation of Hb-content by spectrophotometry in the brain samples using Drabkin's reagent, and **b** estimation of edema volume by wet–dry method, at 24 h post-stroke. Data were expressed as mean ± SD (*n* = 6). Pairs of means with different letters are significantly different, *p* < 0.05

Figure 3a,b (Experiment II) shows the effect of therapies on hemorrhagic transformation and edema. The main effects of RIPerC (*p* = 0.001) and IV-tPA (*p* = 0.0029) on Hb-content were significant such that RIPerC reduced the Hb-content, while IV-tPA increased it. The Hb-content of the eMCAO + RIPerC group was significantly reduced when compared with the eMCAO + Veh group (*p* = 0.031). It was significantly increased in the eMCAO + tPA group compared with eMCAO + Veh (*p* = 0.05), which was decreased when combined with RIPerC therapy in eMCAO + RIPerC + tPA group (*p* = 0.0058). Moreover, RIPerC reduced the edema while it was increased due to late IV-tPA therapy. The main effects of RIPerC (*p* = 0.003) and IV-tPA (*p* = 0.046) on edema were also significant. Edema in the eMCAO + RIPerC group was significantly lower as compared with the eMCAO + Veh group (*p* = 0.048) and the eMCAO + tPA group (*p* = 0.0011). Edema was increased due to late IV-tPA therapy but it was not significantly different as compared with the eMCAO + Veh (*p* = 0.35), possibly due to higher mortality after IV-tPA alone treatment and resulting survival selection bias in the group. There was a significant decrease in the edema volume in eMCAO + RIPerC + tPA group when compared with eMCAO + tPA (*p* = 0.015).

### Combined RIPerC Treatment with IV-tPA Provided Survival Benefits

The survival mortality recorded from experiments I and II had been pooled and presented in Table 1. There was a significant group effect on survival (*p* = 0.0017). RIPerC alone in eMCAO + RIPerC group did not show any significant survival benefits (∼10 % improvement) as compared with the eMCAO + Veh group (76.7 % vs. 66.7 %; *p* = 0.22). Mortality was increased with late IV-tPA alone in the eMCAO + tPA group by ∼20 % as compared with the eMCAO + Veh group (47.5 % vs. 66.7 %; *p* = 0.024). RIPerC therapy prior to IV-tPA increased survival by ∼22 % in eMCAO + RIPerC + tPA group as compared with eMCAO + tPA (70.0 % vs. 47.5 %; *p* = 0.0038) but not in comparison with eMCAO + Veh (*p* = 0.67).

## Discussion

RIPerC therapy after embolic stroke in OVX mice conferred neurobehavioral and neuroprotective benefits. Of clinical importance, RIPerC therapy prior to late IV-tPA treatment reduced the mortality. To the best of our knowledge, this is the first preclinical report in which RIPerC had been tested in OVX females.

In agreement with our previous report in male mice [9], we found that the RIPerC therapy remains effective in improving early CBF and outcomes (Figs. 1 and 2). RIPerC but ‘not’ late IV-tPA alone improved the functional outcomes and reduced the injury size. Contrary to our findings in males [9], IV-tPA therapy alone failed to reduce the injury size in OVX mice, which is in agreement with a previous report [18]. This indicates that OVX mice might have increased risk of early neurovascular stress due to compromised vascular patency and blood brain barrier (BBB) integrity due to surgical ovariectomy [19, 20], which can further limit benefits of IV-tPA treatment. Prior RIPerC therapy reduced the detrimental effects and preserved the benefits of IV-tPA, when combined.

Recanalization by thrombolysis and intra-arterial thrombectomy may not necessarily reperfuse microvessels. Recent preclinical and clinical reports conclude that the improvement in microvascular perfusion is more critical for better post-stroke outcomes [21–23]. As evident from Figs. 1 and 2, only RIPerC but not late IV-tPA-mediated increase in cerebral perfusion reduced the infarct size and improved behavioral outcomes. Recent clinical trial evidence also demonstrates that remote limb conditioning improves cerebral perfusion, improves recovery from stroke, and decreases risk of stroke in patients with intracranial arterial stenosis [24].

Preclinical studies show that gender difference exists after stroke [4, 5]. Stroke is less frequent in pre-menopausal women, but menopause abolishes this advantage and increases the risk of stroke injury. While some preclinical studies used OVX mice 10 days post-ovariectomy [4, 25], we used mice 2-months post-ovariectomy and at 6 months of age. In agreement with the previous reports [26], we noted that 2 months of further aging after ovariectomy eventually brings phenotypic changes in the OVX mice due to significant change in their body weight. Since gain in the body weight after ovariectomy is a very common ovariectomy-associated problem in women [26–28], we assume that 2-months post-OVX mice better models ‘surgical menopause’ than earlier time points. As evident from Fig. 3 and Table 1, RIPerC treatment alone not only attenuated the brain Hb-content as well as edema progression in OVX mice but also reduced the mortality. Late IV-tPA treatment alone resulted in the highest mortality (Table 1) possibly because of higher HT and edema formation. However, edema and injury size in the IV-tPA alone group were not significantly different from the untreated stroke control group, most likely due to survival selection bias. RIPerC when combined with late IV-tPA decreased both Hb-content in the brain as well as edema and reduced the mortality associated with late IV-tPA.

In view of our previous report in male mice [9] and the present communication, RIPerC seems to have a sex independent therapeutic potential for the treatment of stroke. However, the preclinical package still has certain limitations. First, we did not use aged mice of both sexes simultaneously, which better represent the majority of stroke sufferers. We have ongoing work in this direction, which will not only bring further age and sex dependent preclinical information but also the short- and long-term treatment benefits of remote ischemic conditioning. Second, as per the STAIR recommendations, therapeutic potential of RIPerC should be tested in the animals with comorbid conditions like hypertension and diabetes, which further worsen post-stroke outcomes. Third, we detected CBF changes by laser Doppler that mostly measures blood flow close to the brain's surface [21] but not deeper into the brain. It is necessary to further study the effect of RIPerC on CBF after stroke using sensitive techniques and in animals with comorbidities. Collectively, these are caveats in our preclinical package to date and require further attention in future studies.

Despite these limitations, we provide the first evidence that RIPerC is safe and effective in OVX females, a necessary milestone before this therapy can be considered for translation into human stroke and prior to the initiation of clinical trials in acute ischemic stroke.
